# Phase-Dependent MoS_2_ Nanosheets-Embedded Urinary Catheter for Advanced Photothermal Sterilization

**DOI:** 10.3390/ijms27114806

**Published:** 2026-05-26

**Authors:** Muhammad Saukani, Chien-Hung Lai, Dyah Ika Krisnawati, Hsiu-Yi Chu, Andy C. Huang, Tsung-Rong Kuo

**Affiliations:** 1 Department of Mechanical Engineering, Faculty of Engineering, Universitas Islam Kalimantan MAB, Banjarmasin 70124, Indonesia; saukani@uniska-bjm.ac.id; 2 Department of Physical Medicine and Rehabilitation, School of Medicine, College of Medicine, Taipei Medical University, Taipei City 110, Taiwan; chlai@h.tmu.edu.tw; 3 Department of Physical Medicine and Rehabilitation, Taipei Medical University Hospital, Taipei City 110, Taiwan; 4 Taipei Neuroscience Institute, Taipei Medical University, Taipei City 110, Taiwan; 5 Graduate Institute of Biomedical Optomechatronics, College of Biomedical Engineering, Taipei Medical University, Taipei City 110, Taiwan; 6 Department of Nursing, Faculty of Nursing and Midwifery, Universitas Nahdlatul Ulama Surabaya, Surabaya 60237, Indonesia; dyahika@unusa.ac.id; 7 Center for Continuing Care Research (C3R), Universitas Nahdlatul Ulama Surabaya, Surabaya 60237, Indonesia; 8 Graduate Institute of Biomedical Materials and Tissue Engineering, College of Biomedical Engineering, Taipei Medical University, Taipei City 110, Taiwan; d825111001@tmu.edu.tw; 9 Department of Urology, Taipei Medical University Hospital, Taipei City 110, Taiwan; 10 TMU Research Center of Urology and Kidney and Department of Urology, School of Medicine, College of Medicine, Taipei Medical University, Taipei City 110, Taiwan; 11 Graduate Institute of Nanomedicine and Medical Engineering, College of Biomedical Engineering, Taipei Medical University, Taipei City 110, Taiwan; 12 International Ph.D. Program in Biomedical Engineering, College of Biomedical Engineering, Taipei Medical University, Taipei City 110, Taiwan

**Keywords:** 1T-MoS_2_, 2H-MoS_2_, antibacterial, urinary catheter, photothermal therapy, hydrophobicity

## Abstract

The high prevalence of catheter-associated urinary tract infections (CAUTIs) has become a significant concern in the healthcare system, prompting the development of antibacterial urinary catheters to effectively prevent these infections in clinical settings. In this work, metallic phase and semiconducting phase molybdenum disulfide (MoS_2_) embedded polydimethylsiloxane (PDMS) were developed as antibacterial urinary catheters with photothermal sterilization. The metallic phase MoS_2_ (1T-MoS_2_) was synthesized using a facile hydrothermal method, and an annealing process transformed it into the semiconducting phase (2H-MoS_2_). The optical and structural characterizations confirmed the successful preparations of 1T-MoS_2_ nanosheets (1T-MoS_2_ NSs) and 2H-MoS_2_ NSs. The increase in the contents of 1T-MoS_2_ NSs and 2H-MoS_2_ NSs in PDMS resulted in enhanced photothermal conversion, a slight decrease in the water contact angle, and no significant changes in the mechanical properties of the samples. The bacterial growth curves demonstrated the remarkable ability of phase-dependent 1T- and 2H-MoS_2_ NSs-embedded PDMS urinary catheters to inhibit the growth of *E. coli* and *S. aureus* with near-infrared (NIR) laser irradiation. In the agar plate test, exposing PDMS with 0.3% 1T-MoS_2_ or 2H-MoS_2_ to NIR for 10 min demonstrated excellent antibacterial effects, completely eradicating *E. coli* and eliminating over 99.9% of *S. aureus*. The SEM image results highlighted the significant photothermal antibacterial effect of 1T-MoS_2_ PDMS and 2H-MoS_2_ PDMS urinary catheters, effectively damaging and eradicating both *E. coli* and *S. aureus*. The 1T-MoS_2_ PDMS and 2H-MoS_2_ PDMS urinary catheters, with excellent photothermal effects, good hydrophobicity, and superior mechanical properties, demonstrated their potential as photothermal antibacterial catheters for clinical applications.

## 1. Introduction

The Catheter-associated urinary tract infections (CAUTIs) are bacterial infections in the urinary tract that occur in patients who have been catheterized within the previous 48 h [1]. The infection rates are 3 to 5 times higher than those in other hospital patient care areas, with the incidence of CAUTI being 7.78 per 1000 catheter days [2]. CAUTIs are the most common nosocomial infection, accounting for approximately 9% of all healthcare-associated infections, and they can also lead to secondary bloodstream infections [3]. CAUTIs are associated with extended durations of hospitalization and increased mortality. The financial burden of CAUTIs is estimated to vary from USD 603 to 1189 for non-intensive care unit inpatients and up to USD 1764 for intensive care unit patients [4]. *Escherichia coli* (*E. coli*) is a common uropathogen responsible for approximately 75% of CAUTIs, followed by *Pseudomonas aeruginosa*, *Proteus mirabilis*, *Staphylococcus epidermidis*, *Enterococcus faecalis*, and *Klebsiella pneumoniae* [5]. Furthermore, the abiotic surfaces of catheters, which are made of latex, silicone, and polyurethane, are prone to the development of biofilms composed of extracellular polymeric substances, leading to frequent resistance to the penetration of antibiotics [6,7,8]. Successful treatment of biofilms with antibiotics requires concentrations that are 1500 times higher than standard doses, which can result in bacterial resistance and toxicity [9]. Therefore, numerous strategies to reduce the risk of CAUTIs have been developed through clinical and technological approaches, including aseptic catheter insertion techniques, optimal catheter exchange timing, antibiotic prophylaxis, and modifications of catheter materials and coatings [10].

Modifications of catheter materials and coatings have the potential to provide complete protection against CAUTIs by creating catheters with antimicrobial and antifouling properties [11]. Generally, antifouling catheters utilize material coatings, including hydrophilic polymers, amphiphilic polymers, superhydrophobic polymers, hydrogels, polymer brushes, and polyzwitterions [12]. This strategy aims not to kill bacteria but to prevent bacterial biofilms from attaching to susceptible surfaces of catheters through steric repulsion, electrostatic repulsion, and low surface energy. Another approach to combat bacterial biofilms on catheter surfaces involves coating them with biocidal materials. Various mechanisms to counter bacterial biofilms include the release of antimicrobial compounds (such as antiseptics, antibiotics, antimicrobial peptides, and metal nanoparticle (NP)-based materials), contact killing (using antibiotics, metal-based NPs, antimicrobial peptides, carbon materials, and biopolymers), and the disruption of biofilm architecture by quorum-sensing inhibitors [13,14,15,16,17]. Metal-based NPs are popular antibacterial agents for combating resistant bacteria, including bacterial biofilms. The ability to control their size and shape enhances their antibacterial performance by inducing membrane disruption, direct contact, and reactive oxygen species (ROS) production [18,19,20,21,22]. Among NPs, silver NPs are one of the few microbial agents approved by the U.S. Food and Drug Administration (FDA) [23]. However, due to challenges with controlling ion release kinetics and low selectivity, silver NPs exhibit low biocompatibility with mammalian cells. Therefore, replacing them with biocompatible nanomaterials would be the best option for developing an antibacterial catheter.

Molybdenum disulfide (MoS_2_) is a biocompatible material that can be synthesized via a top-down approach, like plasma exfoliation and alkali metal intercalation, or a bottom-up approach using chemical vapor deposition (CVD) and hydrothermal methods [24,25,26,27]. MoS_2_ has two main phases: a metallic phase (1T-MoS_2_) and a semiconducting phase (2H-MoS_2_), with the 2H-phase being thermodynamically stable in bulk [28,29,30]. Through plasma exfoliation and the Li intercalation method, 1T-MoS_2_ can be synthesized from bulk MoS_2_ and revert to the initial phase after annealing at 200 °C in an Ar atmosphere. In our previous work, 1T-MoS_2_ nanosheets (NSs) were synthesized using a simple hydrothermal method, and the resulting 1T-MoS_2_ NSs were then annealed at 300 °C to obtain 2H-MoS_2_ NSs [30,31]. This method is more efficient compared to CVD methods, which require a growth temperature range of 780 °C to 800 °C to synthesize 1T-MoS_2_.

Due to its excellent properties, MoS_2_ has a wide range of potential applications, including as an antibacterial agent, an energy storage material, and a photocatalyst for hydrogen evolution reactions, organic pollutant degradation, and CO_2_ reduction [32,33,34,35,36,37,38,39]. As an antibacterial agent, the bacterial eradication mechanism of MoS_2_ involves not only ion release that generates reactive oxygen species (ROS) or direct contact with bacterial cell walls, causing membrane disruption, but also the use of external triggers to enhance antibacterial activity [40,41,42,43,44]. External stimuli applied to MoS_2_ have been shown to accelerate bacterial eradication. Visible light can enhance ROS generation, while near-infrared (NIR) light can enhance heat generation, leading to bacterial death through a hyperthermic mechanism [45,46,47,48,49]. Although pristine MoS_2_ exhibits a broad ability to combat both Gram-negative and Gram-positive bacterial strains, to our knowledge, none of the reports have explored the use of these materials for antibacterial catheters, whether by coating or material modification.

In this work, two main phases of MoS_2_, 1T-MoS_2_ NSs and 2H-MoS_2_ NSs, were synthesized as antibacterial agents using a simple hydrothermal method. 1T-MoS_2_ NSs and 2H-MoS_2_ NSs were characterized using X-ray diffraction (XRD), X-ray photoelectron spectroscopy (XPS), Raman spectroscopy, ultraviolet-visible (UV-Vis) spectroscopy, scanning electron microscopy (SEM), transmission electron microscopy (TEM), and high-resolution transmission electron microscopy (HR-TEM). The catheters were developed by the use of silicon-based polydimethylsiloxane (PDMS) embedding with 1T-MoS_2_ NSs (1T-MoS_2_@PDMS) and 2H-MoS_2_ NSs (2H-MoS_2_@PDMS). The antibacterial performances of the 1T-MoS_2_@PDMS and the 2H-MoS_2_@PDMS were investigated through a photothermal effect using near-infrared (NIR) irradiation. Numerous characterizations of the 1T-MoS_2_@PDMS and the 2H-MoS_2_@PDMS were examined in this paper, including photothermal property, water contact angle, mechanical property, antibacterial performance, and biocompatibility.

## 2. Results

### 2.1. Material Characterizations of 1T-MoS_2_ NSs and 2H-MoS_2_ NSs

**Figure 1:**
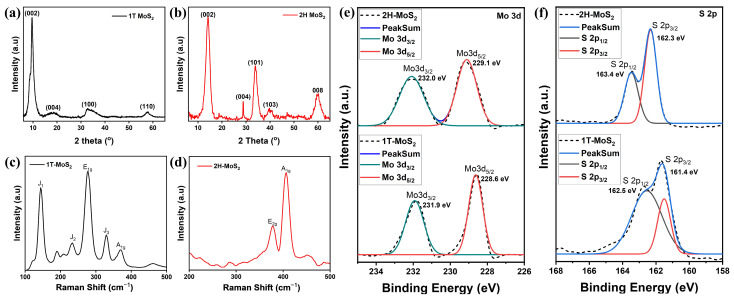
X-ray diffraction patterns of (**a**) 1T-MoS_2_ NSs and (**b**) 2H-MoS_2_ NSs. Raman spectra of (**c**) 1T-MoS_2_ NSs and (**d**) 2H-MoS_2_ NSs. (**e**) XPS spectra of Mo 3d for 1T-MoS_2_ NSs and 2H-MoS_2_ NSs. (**f**) XPS spectra of S 2p for 1T-MoS_2_ NSs and 2H-MoS_2_ NSs.

The MoS_2_ polymorph depends on the coordination of the sulfur atoms and the stacking order of the layers, which changes the symmetry of the sulfur atoms in the structure [50]. XRD data were used to analyze the crystalline structures of 1T-MoS_2_ NSs and 2H-MoS_2_ NSs. The diffractogram of the hydrothermally synthesized material is exhibited in Figure 1a with crystalline planes (002), (004), (100), and (110), appearing at 9.3°, 18°, 33°, and 57.5°, respectively. The peaks of the crystalline plane represent 1T-MoS_2_ NSs, the metastable phase with an octahedral structure [30]. This led to the orientation of the crystal structure. The annealing process at 300 °C for 2 h caused a 2θ shift in Figure 1b, indicating the crystal structure’s reorientation. The 2θ positions changed to become 14.38°, 28.79°, 33.73°, 39.69°, and 59.63°, which respectively reflect the (002), (004), (101), (103), and (008) crystalline planes. This indicated that a structural transformation had occurred from 1T-MoS_2_ NSs to 2H-MoS_2_ NSs [51,52,53]. The quantitative analysis by Highscore Plus yielded a single phase of 2H MoS_2_ NSs with a hexagonal structure (JCPDS 37-1492). Raman spectroscopic features were used to confirm the distinct crystalline structure and the successful transformation of 1T-MoS_2_ NSs to 2H-MoS_2_ NSs after the annealing process. Low-energy Raman spectra of the samples synthesized by a hydrothermal method are shown in Figure 1c. Peaks of Raman shifts existed at 146, 234, 277, 329, and 372 cm^−1^, which were associated with phonon modes J_1_, J_2_, E_1g_, J_3_, and A_1g_, respectively. The peak position refers to the successful synthesis of the 1T-MoS_2_ phase [28]. After the annealing process, the Raman spectrum in Figure 1d only shows two prominent peaks of Raman shifts at 379 cm^−1^ (E_2g_) attributed to the longitudinal acoustic phonon mode S–Mo–S and at 405 cm^−1^ (Ag) corresponding to out-of-lane vibration S atoms [46]. The disappearance of the J_1_, J_2_, and J_3_ peaks, which are phonon modes, after the annealing process indicates the conversion of the metal phase (1T-MoS_2_) to a semiconductor phase (2H-MoS_2_). XPS characterization was used to distinguish the phase of MoS_2_ NSs, confirming the successful synthesis of 1T-MoS_2_ NSs and 2H-MoS_2_ NSs. The survey spectra of the overall electronic states of the 1T-MoS_2_ NSs and 2H-MoS_2_ NSs samples are presented in Figure S1a,b, while the high-resolution Mo 3d and S 2p spectra are demonstrated in Figure 1e,f, respectively. The XPS data supported the prior characterization, with Mo 3d spectra at binding energies of ~228.8 and ~232.0 eV corresponding to Mo^4+^ 3d_5/2_ and Mo^4+^ 3d_3/2_, respectively. This result is consistent with the previous reports [54,55]. After annealing, these peaks shift toward higher binding energy (~229.1 and ~232.0 eV), indicating a phase transformation from 1T to 2H-MoS_2_. Further evidence is provided by the S 2p spectra, which reveal binding energies of ~161.4 and ~162.5 eV corresponding to S 2p_3/2_ and S 2p_1/2_. Following annealing, these peaks shift to ~162.3 and ~163.4 eV. This phenomenon is attributed to the decreased electron density around the Mo and S atoms, consistent with the structural transition from the metallic 1T phase to the semiconducting 2H phase.

**Figure 2:**
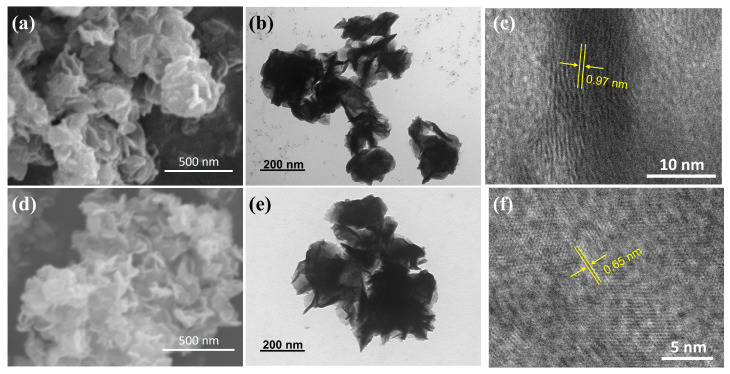
Morphology of 1T-MoS_2_ NSs under (**a**) SEM, (**b**) TEM, and (**c**) HR-TEM. Morphology of 2H-MoS_2_ NSs under (**d**) SEM, (**e**) TEM, and (**f**) HR-TEM.

The XRD, Raman, and XPS spectral data confirmed that 1T-MoS_2_ and 2H-MoS_2_ were successfully synthesized by a straightforward technique. The morphologies of both samples were characterized by SEM, TEM, and HR-TEM images. The flower-like morphology consisted of an MoS_2_ NSs, as shown in Figure 2a,d by SEM images. TEM images in Figure 2b,e confirm the multi-sheet constructed flower-like morphology of MoS_2_. The average sizes of the 1T-MoS_2_ NSs and 2H-MoS_2_ NSs flower-like particles were calculated to be approximately 400–800 nm. In addition, EDX data (spectra and mapping) are presented in Figure S2 (1T-MoS_2_ NS) and Figure S3 (2H-MoS_2_ NSs), and the elemental quantitative analysis calculated by EDX is demonstrated in Table S1 (1T-MoS_2_ NS) and Table S2 (2H-MoS_2_ NSs). EDX data show the percentages of sulfur (S) at 62.69% and of molybdenum (Mo) at 37.31% for 1T-MoS_2_ NSs, while 2H-MoS_2_ NSs contained 61.25% S and 38.75%. Moreover, HR-TEM was performed to obtain structural information. The measured interplanar spacing of ~0.97 nm in Figure 2c can be assigned to the (002) plane of 1T-MoS_2_, which is consistent with previously reported values for 1T-MoS_2_ nanosheets (~0.95 nm) [30,56]. The slightly larger spacing observed in this work is attributed to the loosely stacked nanosheet structure, which may lead to interlayer expansion, as commonly observed in hydrothermally synthesized MoS_2_. After annealing, the interplanar spacing decreases to ~0.65 nm, indicating more ordered, compact stacking in the 2H-MoS_2_ phase and confirming the structural transformation from trigonal (P3m1) to the hexagonal (P63/MMC) [30,56].

**Figure 3:**
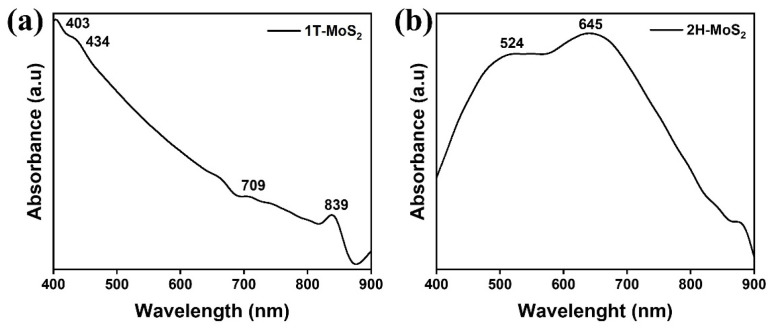
UV-Vis spectra of (**a**) 1T-MoS_2_ NSs and (**b**) 2H-MoS_2_ NSs.

UV-Vis spectra of 1T-MoS_2_ NSs and 2H-MoS_2_ NSs dispersed in acetone are shown in Figure 3. 1T-MoS_2_ spectra in Figure 3a exhibit a broad absorption profile without distinct excitonic features, which indicates the absence of stable exciton formation. This characteristic is attributed to the metallic nature of the 1T phase, in which a high density of free electrons induces Coulombic screening, thereby suppressing electron-hole and excitonic transitions [31]. In contrast with the UV-Vis spectra in Figure 3b, which exhibited an absorption peak at ~645 nm, corresponding to an A excitonic transition, along with a shoulder feature at ~524 nm that may be associated with the B excitonic transition, which originates from a direct interband transition at the K point of the Brillouin zone due to spin-orbit splitting in the valence band. The presence of an excitonic feature indicates a well-defined band structure characteristic of a semiconductor [57].

### 2.2. Characterizations of MoS_2_ Nanosheets-Embedded Urinary Catheters

**Figure 4:**
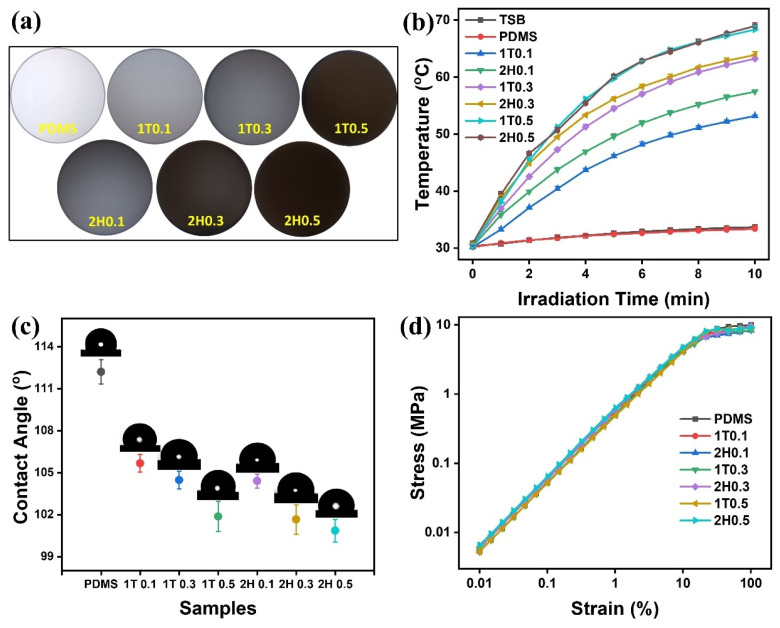
(**a**) Optical images, (**b**) photothermal characteristics, (**c**) contact angles, and (**d**) stress–strain curve characteristics of PDMS, 1T0.1, 1T0.3, 1T0.5, 2H0.1, 2H0.3, and 2H0.5 samples.

Embedding 1T-MoS_2_ and 2H-MoS_2_ NSs in PDMS as a bulk material offers several advantages over surface coating. Bulk embedding ensures a uniform distribution of nanosheets throughout the catheter, providing consistent photothermal and antibacterial properties across the entire structure, even if the surface layer is damaged or worn over time. Additionally, it enhances the mechanical stability and durability of the catheter, as the active materials are integrated into the matrix rather than relying on surface adhesion. This approach also minimizes the risk of nanosheet detachment, ensuring long-term functionality and safety, which are critical for biomedical applications. Therefore, the catheter model was produced by embedding 1T-MoS_2_ NSs and 2H-MoS_2_ NSs in PDMS as the raw material. Images of a pristine PDMS urinary catheter and PDMS with different MoS_2_ contents were taken, as shown in Figure 4a. Hexane can induce swelling of PDMS; therefore, its presence was effective in assisting the dispersion of nanomaterials into PDMS [58,59]. It can be seen from those figures that pristine PDMS exhibited a transparent color, and PDMS samples with increasing MoS_2_ contents turned darker. The 2H-MoS_2_ NSs provided a darker effect than the 1T MoS_2_ NSs content. 1T-MoS_2_ and 2H-MoS_2_ have different phases, namely metal and semiconductor, respectively. The biocompatibility assessment using Vero cells confirmed that PDMS, 1T-MoS_2_@PDMS, and 2H-MoS_2_@PDMS support normal cell proliferation and exhibit excellent biocompatibility (Figure S4). Both materials were used as photothermal agents by combining them with PDMS. The relationship between time exposure of the NIR laser (1.5 W/cm^2^) and heat generation by the sample with different contents of MoS_2_ in 1.5 mL of the TSB solution is shown in Figure 4b. The temperature needed to kill bacteria ranged from 55–60 °C for *E. coli* and 60–65 °C for *S. aureus* [60,61]. The smallest concentration (0.1%) of either 1T-MoS_2_ NSs or 2H-MoS_2_ NSs was embedded in PDMS and then exposed to the NIR laser for 10 min, generating respective final temperatures of 53.2 ± 0.2 and 57.4 ± 0.1 °C. The other samples (1T0.3, 2H0.3, 1T0.5, and 2H0.5) reached the eradication temperature after being irradiated by NIR for less than 5 min. After 10 min of NIR laser irradiation, the respective final temperatures of the 1T0.3, 2H0.3, 1T0.5, and 2H0.5 samples were 63.2 ± 0.2, 63.9 ± 0.5, 68.4 ± 0.6, and 68.9 ± 0.5 °C. The photothermal performance of the 2H0.5 urinary catheter in artificial urine and TSB showed similar results in both conditions, demonstrating its efficacy under relevant simulated environments (Figure S5). Moreover, the photothermal conversion efficiencies of 1T01, 1T03, 1T05, 2H01, 2H03, and 2H05 were respectively calculated to be 40.24, 45.38, 41.32, 45.35, 43.49, and 42.98% [62,63,64]. (see Figure S6 in Supporting Information) Water contact angles of PDMS and PDMS embedded with 1T-MoS_2_ NSs or 2H-MoS_2_ NSs are depicted in Figure 4c. The contact angle of PDMS decreased after MoS_2_ NSs incorporation. Pristine PDMS displayed hydrophobic properties with a contact angle of 112.4° ± 0.3°, leading to the undesired adsorption of proteins [65]. These phenomena affect the transportation of analytes and reduce the performance of separation techniques and the sensitivity of detection methods [66,67]. The effect of embedding MoS_2_ NSs in PDMS to improve the surface quality was to decrease the water contact angle, becoming 101.8° ± 0.7° for 1T and 100.9° ± 0.5°. The impact of MoS_2_ NSs addition to PDMS validated that its incorporation could improve the hydrophilicity of PDMS, which would ease intubation in the body [66]. The mechanical properties are represented by the stress–strain curves in Figure 4d. Catheters require materials with adequate flexibility and mechanical strength to ensure safe and reliable performance during use. The addition of 1T-MoS_2_ NSs or 2H-MoS_2_ NSs to PDMS urinary catheters did not significantly affect the Young’s modulus, indicating that the 1T-MoS_2_ and 2H-MoS_2_ PDMS urinary catheters retain the inherent elasticity of the original PDMS material. The maximum elongation of approximately 21% and the maximum load upon rupture of around 7.8 MPa demonstrate the ability of the 1T-MoS_2_ and 2H-MoS_2_ PDMS urinary catheters to withstand mechanical deformation and stress during catheter insertion and operation. These stress–strain curve results confirm the suitability of 1T-MoS_2_ and 2H-MoS_2_ PDMS composites for catheter applications, providing a balance of flexibility and strength under physiological conditions.

### 2.3. Antibacterial Performance of Phase-Dependent MoS_2_ Nanosheets-Embedded Urinary Catheters

**Figure 5:**
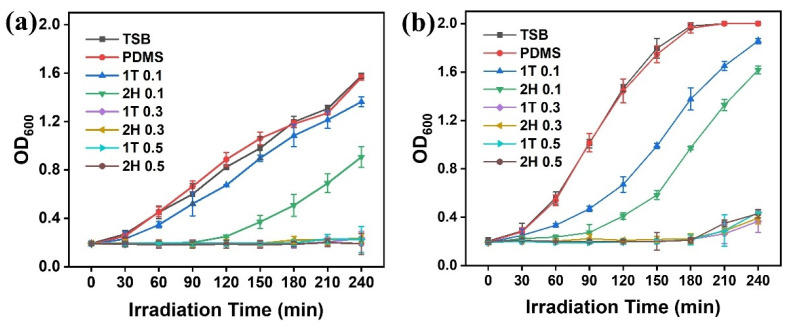
Growth curves of (**a**) *E. coli* and (**b**) *S. aureus* after irradiation with NIR laser for 10 min.

Based on photothermal data in Figure 4b, after NIR laser irradiation of PDMS, 1T0.1, 2H0.1, 1T0.3, 2H0.3, 1T0.5, and 2H0.5 samples for 10 min, respective final temperatures were 33.4 ± 0.6, 53.2 ± 0.2, 57.4 ± 0.1, 63.2 ± 0.2, 63.9 ± 0.5, 68.4 ± 0.6, and 68.9 ± 0.5 °C. Those data were used for reference to observe growth curves of a Gram-positive bacterium (*S. aureus*) and a Gram-negative bacterium (*E. coli*). Samples were put into 1.5 mL of a bacterial suspension (OD_600_ = 0.2) after being exposed to 808-nm NIR for 10 min, and then growth curves of bacteria at 37 °C were recorded (Figure 5). Growth curves of bacteria of the control (TSB) and PDMS samples showed a gradual increase for both bacteria. The growth of *S. aureus* was faster than that of *E. coli* because *S. aureus* is a mesophilic bacterium that grows best at moderate temperatures in the human body at 37 °C, while *E. coli* is a thermophilic bacterium that grows best at higher temperatures (37–40 °C) [68]. The growth curves of bacteria in Figure 5a show a remarkable ability of 1T0.5 and 2H0.5 to inhibit *E. coli* growth until 240 min, while *S. aureus* (Figure 5b) demonstrated bacterial growth starting after 180 min. These results indicate that *S. aureus* had a faster reproductive ability than *E. coli*, even though some bacteria died. The outer membrane of *E. coli* becomes reversibly disrupted at a temperature of >46 °C [69]. Higher temperatures can inhibit the proliferation and mobility of bacteria through membrane damage and autolysis. As reported, temperatures higher than 60 °C cause bacterial death [61]. After 10 min, NIR irradiation could not restrain the growth of bacteria due to the final temperature being less than 55 °C, with both bacteria beginning to grow in less than 30 min.

**Figure 6:**
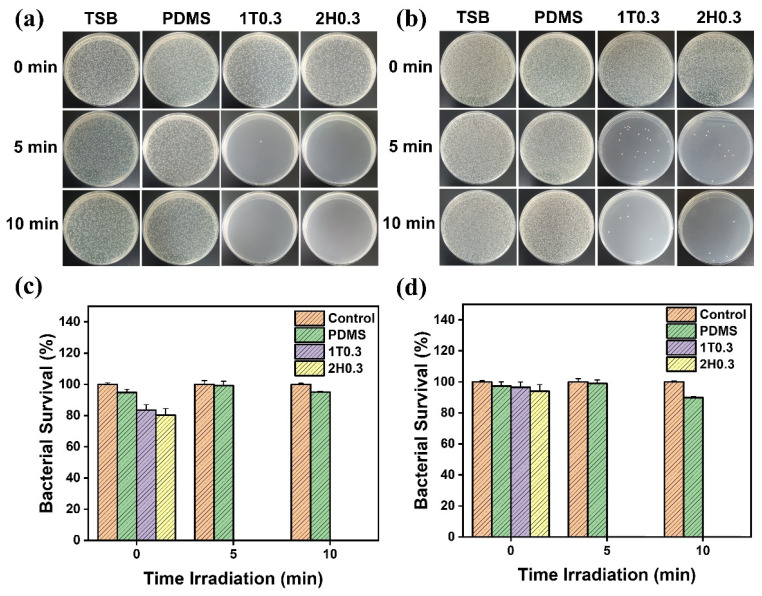
Colonies of bacteria after incubation with the catheter model and treatment with different irradiation times for (**a**) *E. coli* and (**b**) *S. aureus*. Bacterial survival values with laser irradiation times of 0, 5, and 10 min were measured for various samples of (**c**) *E. coli* and (**d**) *S. aureus*.

Based on the photothermal properties, water contact angle, stress–strain curve, and growth curve of bacteria, 0.3% MoS_2_ in PDMS was sufficient for further examination. Figure 6a (*E. coli*) and Figure 6b (*S. aureus*) show colonies of bacteria on the agar plate after 24 h with different irradiation times. To clearly define the antibacterial performance, bacterial survival was determined by Equation (1), and results are displayed in Figure 6c (*E. coli*) and Figure 6d (*S. aureus*). In Figure 6c, for control (TSB) and PDMS, there is no significant bacterial eradication with 5 and 10 min of NIR laser irradiation. The 1T0.3 exhibited excellent performance; after 5 min of irradiation, 0.007% of *E. coli* remained, and after 10 min, no colony could grow. The 2H0.3 shows the complete eradication of *E. coli* after exposure for both 5 and 10 min. Figure 6d displays bacterial survival of *S. aureus* after being treated for different irradiation times. Exposure times to the NIR laser of 5 and 10 min with either 1T0.3 or 2H0.3 were insufficient to kill all *S. aureus*; however, they were effective in inhibiting bacterial division. From those data, 10 min of irradiation resulted in 0.032% (1T0.3) and 0.025% (2H0.3) bacterial survival. The killing rate of *S. aureus* was lower than that of *E. coli* because the thicker cell walls of the Gram-positive bacterium *S. aureus* provide better defense against external treatment compared to *E. coli* [70].

**Figure 7:**
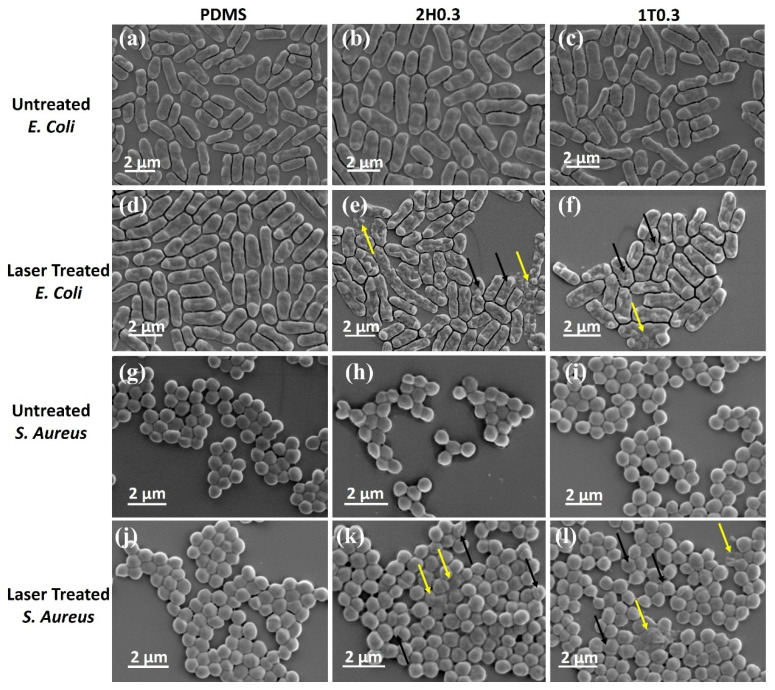
Bacterial morphologies of *E.coli* without laser irradiation treatment of (**a**) PDMS, (**b**) 2H0.3, and (**c**) 1T0.3 samples. Bacterial morphologies of *E. coli* under laser treatment irradiation for 5 min of (**d**) PDMS, (**e**) 2H0.3, and (**f**) 1T0.3 samples. Bacterial morphologies of *S. aureus* without laser irradiation treatment of (**g**) PDMS, (**h**) 2H0.3, and (**i**) 1T0.3 samples. Bacterial morphologies after laser irradiation for 5 min of (**j**) PDMS, (**k**) 2H0.3, and (**l**) 1T0.3 samples. Black arrow: bacterial cell wall damage. Yellow arrow: complete bacterial destruction.

Bacterial morphologies of *E. coli* and *S. aureus* after being incubated with PDMS, 1T0.3, and 2H0.3 for 4 h were observed by SEM, as shown in Figure 7. Without laser irradiation treatment, the morphology of *E. coli* exhibited a smoother surface with an integral membrane structure (Figure 7a–c). These results confirmed that PDMS, 1T0.3, and 2H0.3 exhibited no significant antibacterial effect without laser irradiation. Although NIR was applied to PDMS, a different morphology was not seen in untreated samples. In contrast, the morphology of *E. coli* in the 1T0.3 and 2H0.3 samples showed differences compared to the untreated samples, as shown in Figure 7e,f. A hyperthermic effect was shown to have a severe damaging effect in Figure 7e,f, with bacterial cell wall damage (black arrow) and complete bacterial destruction (yellow arrows). Interestingly, the photothermal treatment of PDMS containing MoS_2_ for an antibacterial catheter was shown to be an effective way to eradicate bacteria, where all the developed steps and states effectively killed the bacteria. *S. aureus* is a Gram-positive strain of bacteria with a thicker peptidoglycan than *E. coli*, which is more challenging to obtain total eradication [71]. In addition, morphologies of *S. aureus* with and without laser irradiation are shown in Figure 7g–l. A similar condition with *E. coli*, without laser irradiation with 2H0.3 and 1T0.3, showed a smooth surface and good membrane integrity. After applying laser irradiation for 5 min, the photothermal effect was destructive to *S. aureus*. As seen in the apparent destruction of *S. aureus*, membrane leakage and rupture of the structural integrity of *S. aureus* caused bacterial death. Nevertheless, bacterial structure without defects still existed, even though it was subjected to the photothermal effect, because of the thick peptidoglycan layer, which protected against external stress. Overall, these findings demonstrate the significant photothermal antibacterial effect of urinary catheter PDMS containing different phases of MoS_2_, which effectively damages and eradicates both *E. coli* and *S. aureus*. However, the thicker peptidoglycan layer of *S. aureus* presents greater resistance, highlighting the challenge of achieving complete bacterial eradication in Gram-positive strains.

## 3. Discussion

This study demonstrates the successful synthesis and structural transformation of MoS_2_ NSs from the 1T to the 2H phase through hydrothermal synthesis and subsequent annealing. Structural characterization using XRD, Raman spectroscopy, and XPS confirmed this transition, highlighting the distinct crystalline and electronic properties of the two phases. The XRD data revealed a shift in 2θ positions after annealing, indicating a reorientation of the crystal structure from the metastable 1T phase (octahedral structure) to the stable 2H phase (hexagonal structure). Raman spectra further corroborated this transformation, with the disappearance of phonon modes (J_1_, J_2_, and J_3_) specific to the metallic 1T phase and the emergence of peaks corresponding to the semiconducting 2H phase (E_2g_ and A_1g_ modes). Additionally, XPS analysis showed a binding energy shift of ~0.8 eV, reflecting reduced electron density, consistent with the phase transition. Morphological analysis using SEM and TEM revealed a flower-like structure for both 1T and 2H MoS_2_ NSs, with particle sizes ranging from 400 to 800 nm. The HR-TEM analysis showed a reduction in d-spacing from 0.97 nm (1T phase) to 0.65 nm (2H phase), confirming the structural transformation. Elemental analysis using EDX revealed a consistent sulfur-to-molybdenum ratio, demonstrating the chemical stability of MoS_2_ during the phase change. UV-Vis spectroscopy further highlighted the optical properties of the two phases. The 1T phase exhibited excitonic peaks within the NIR region, while the 2H phase showed characteristic absorption peaks at 524 and 645 nm. These results indicate that both phases exhibit tunable optical properties, making them suitable for applications such as antibacterial treatments, where UV and NIR stimulation can enhance performance. Overall, the systematic synthesis and characterization of 1T and 2H MoS_2_ NSs provide a promising foundation for their application in various fields, particularly in optoelectronics and biomedicine.

Furthermore, this study highlights the integration of 1T-MoS_2_ and 2H-MoS_2_ NSs into PDMS to fabricate urinary catheters with enhanced photothermal, hydrophilic, and mechanical properties. Both 1T-MoS_2_ (metallic phase) and 2H-MoS_2_ (semiconducting phase) were embedded into PDMS, resulting in visually darker samples with increasing MoS_2_ content, with 2H-MoS_2_ producing a more pronounced darkening effect. As photothermal agents, both 1T-MoS_2_ and 2H-MoS_2_ demonstrated efficient heat generation under NIR laser exposure, achieving bacterial eradication temperatures within 5–10 min. Notably, 2H-MoS_2_ exhibited superior photothermal performance, with the 2H0.5 sample reaching a maximum temperature of 68.9 ± 0.5 °C, making it more effective for antibacterial applications. While photothermal conversion efficiency calculations were performed and discussed, it is critical to note that these calculations are not equivalent to conducting a direct biological thermal-only control experiment. Future studies should aim to include a dedicated thermal-only control experiment to assess the biological effects of heat in isolation. This would allow for a more comprehensive understanding of the contributions of thermal effects versus those mediated by the photothermal agent. Despite this limitation, the results presented here provide valuable insights into the photothermal properties and their potential applications, laying the groundwork for further exploration.

The incorporation of MoS_2_ NSs also improved the hydrophilicity of PDMS, as evidenced by a reduction in the water contact angle from 112.4° to approximately 100°. This enhanced hydrophilicity is beneficial for reducing protein adsorption and improving ease of intubation. Furthermore, mechanical testing demonstrated that the addition of MoS_2_ NSs did not compromise the flexibility or strength of PDMS catheters. The stress–strain curves confirmed sufficient elongation (21%) and rupture strength (7.8 MPa), ensuring reliable performance during catheter use. The results validate the potential of MoS_2_-embedded PDMS catheters for biomedical applications, offering a combination of effective antibacterial properties, improved surface quality, and mechanical durability.

The photothermal antibacterial performance of PDMS combined with 1T-MoS_2_ and 2H-MoS_2_ NSs has been explored for its potential application in urinary catheter materials. The photothermal data revealed that samples with 0.3% (1T0.3 and 2H0.3) and 0.5% (1T0.5 and 2H0.5) MoS_2_ NSs achieved temperatures above 60 °C under 808-nm NIR laser irradiation for 10 min, sufficient to eradicate bacteria. The clinical use of photothermal antibacterial systems like PDMS@MoS_2_ urinary catheters relies on practical strategies, with external NIR irradiation being the most effective and feasible due to its non-invasive activation of MoS_2_ NSs to kill bacteria, ease of application during routine care, and minimal risk of complications. However, samples with 0.1% MoS_2_ content (1T0.1 and 2H0.1) reached temperatures below 55 °C and were less effective in inhibiting bacterial growth. These findings suggest that 0.3% MoS_2_ in PDMS is optimal for antibacterial applications due to its balance of efficacy and material efficiency. Growth curve analyses showed that 1T0.5 and 2H0.5 effectively inhibited *E. coli* growth for up to 240 min, while *S. aureus* growth resumed after 180 min due to its faster reproductive ability. The thicker peptidoglycan layer in *S. aureus* provides greater resistance compared to the thinner outer membrane of Gram-negative *E. coli*. This was further supported by bacterial survival data, where 1T0.3 and 2H0.3 eradicated *E. coli* colonies after 10 min of irradiation, while only 0.032% (1T0.3) and 0.025% (2H0.3) of *S. aureus* survived. The lower killing rate of *S. aureus* highlights the challenge of eradicating Gram-positive bacteria due to their structural defenses.

While this study successfully demonstrates the antibacterial efficacy of the MoS_2_ PDMS urinary catheters under NIR irradiation, it does not evaluate the potential impact of NIR irradiation on healthy cells and tissues in the surrounding area. The absence of such data makes it difficult to fully assess the safety and clinical applicability of this approach. Addressing this limitation will require future studies to systematically investigate cell compatibility under clinically relevant NIR irradiation conditions. Moreover, the evaluation of temperature distribution around the catheter and surrounding tissues during NIR irradiation is crucial for ensuring tissue safety and minimizing potential thermal damage. Future studies should focus on mapping thermal gradients to confirm that heat generation remains localized to the target area. Additionally, the use of dosimetry and real-time temperature monitoring will be essential for optimizing irradiation parameters, enabling precise thermal control, and ensuring the safety of surrounding healthy tissues. These measures are vital for facilitating the safe and effective clinical translation of the MoS_2_ PDMS urinary catheters.

SEM images confirmed that photothermal treatment caused severe damage to bacterial cells. Without NIR irradiation, the bacterial morphology remained intact, indicating that PDMS without photothermal activation had no significant antibacterial effect. After NIR exposure, *E. coli* cells treated with 1T0.3 and 2H0.3 exhibited disrupted membranes and complete structural destruction. Similarly, *S. aureus* cells showed membrane leakage and damage, although some intact structures persisted due to their thicker cell walls. Overall, the addition of MoS_2_ NSs to PDMS improves its ability to generate heat under NIR irradiation, effectively eliminating bacteria in the process. The 2H-MoS_2_ phase exhibited slightly better antibacterial performance than the 1T-MoS_2_ phase. These findings demonstrate the potential of MoS_2_-embedded PDMS as an effective material for urinary catheters, with promising applications in antibacterial treatments. However, the resistance of *S. aureus* highlights the need for further optimization to ensure the complete eradication of Gram-positive bacteria.

## 4. Materials and Methods

### 4.1. Materials

Molybdic acid (H_2_MoO_4_, ≥85%) and thiourea (SC(NH_2_)_2_, 99%) were purchased from Acros Organic (Morris, NJ, USA). Trypticasein soy broth (TSB) was purchased from Condalab (Madrid, Spain). Bacteriological-grade agar and phosphate-buffered saline (PBS) were purchased from Bioman Scientific (Taipei, Taiwan). LB broth Miller was purchased from BioShop (Burlington, ON, Canada). The Sylgard 184 silicon elastomer, including a base and curing agent, was obtained from Dow Silicones (Midland, MI, USA). Ethanol and n-hexane were obtained from Merck (Darmstadt, Germany). Dulbecco’s modified Eagle medium (DMEM), fetal bovine serum (FBS), and Invitrogen calcein AM solution were obtained from ThermoFisher Scientific (Carlsbad, CA, USA).

### 4.2. Preparations of 1T-MoS_2_ NSs and 2H-MoS_2_ NSs

The metallic phase of 1T-MoS_2_ NSs and the semiconducting phase of 2H-MoS_2_ NSs were synthesized using a hydrothermal method, based on previous literature, with certain modifications [30]. To prepare 1T-MoS_2_ NSs, 0.005 M molybdic acid and 0.0125 M thiourea were mixed together in a Teflon container, and 40 mL of deionized (DI) water was added. The mixture was stirred at 500 rpm at room temperature for 40 min, and the complete reaction was marked by a change in the color of the solution from white to transparent. Afterward, the Teflon container was placed into a hydrothermal reactor and heated to 180 °C for 24 h. The solution was poured into a 50-mL centrifuge tube and then centrifuged at 7500 rpm for 10 min to segregate the supernatant and precipitate. The precipitate was washed (with DI water and ethanol) and then dried at 65 °C for 24 h. 1T-MoS_2_ NSs powder (dry precipitate product) was kept in a dry box for further investigation. To acquire 2H-MoS_2_ NSs, 1T-MoS_2_ NSs powder was annealed in an inert gas (N_2_) atmosphere at 300 °C for 2 h. The resulting 2H-MoS_2_ NSs were kept in a dry box for the following experiment.

### 4.3. Characterizations of 1T-MoS_2_ NSs and 2H-MoS_2_ NSs

To confirm the successful syntheses of 1T-MoS_2_ NSs and 2H-MoS_2_ NSs, several characterizations were conducted by XRD, XPS, Raman spectroscopy, UV-Vis spectroscopy, SEM, SEM-energy dispersive X-ray (SEM-EDX), TEM, and HR-TEM. The phase and crystal structure of MoS_2_ were identified with a X-ray diffractometer (D2 Phaser, Bruker, Billerica, MA, USA). The chemical composition and oxidation state of MoS_2_ were determined by XPS (ESCALAB Xi, ThermoFisher, Waltham, MA, USA). The morphology of MoS_2_ was recorded by SEM (SU3500, Hitachi, Tokyo, Japan), SEM-EDX (Quantax EDS, Bruker, Billerica, MA, USA), TEM (HT7700, Hitachi, Tokyo, Japan), and HR-TEM (JEM-2100, JEOL, Tokyo, Japan). The optical properties were recorded by UV-Vis (V-770, Jasco, Tokyo, Japan) and Raman spectroscopy (UniDRON, CLT, New Taipei City, Taiwan).

### 4.4. Preparation of 1T-MoS_2_@PDMS and 2H-MoS_2_@PDMS Urinary Catheters

A urinary catheter was created by combining PDMS (Sylgard 184 silicon elastomer, Midland, MI, USA) and MoS_2_ (1T and 2H phases). Pristine PDMS was prepared as a control sample. To prepare the 1T-MoS_2_@PDMS urinary catheter, PDMS was doped with various weight percentages of 1T-MoS_2_, including 0.1% (1T0.1), 0.3% (1T0.3), and 0.5% (1T0.5). The PDMS was also doped with various weight percentages of 2H-MoS_2_, including 0.1% (2H0.1), 0.3% (2H0.3), and 0.5% (2H0.5). For the experiments, various weights of MoS_2_ powders (1T-MoS_2_ and 2H-MoS_2_) were mixed with 0.72 mL of n-hexane and sonicated for 10 min at 25 °C to disperse the powder in the solution. Sylgard base (10 g) and 1 g of curing agent were then added to the solution and stirred at room temperature for 1.5 h until homogeneous. The solution was cast into a glass Petri dish (10 cm in diameter) and then degassed for 1 h to remove bubbles in a furnace vacuum oven at 40 °C. Afterward, the temperature was increased to 70 °C for 12 h. The final thickness of 1T-MoS_2_@PDMS and 2H-MoS_2_@PDMS catheters was around 2 mm.

### 4.5. Photothermal Characteristics of MoS_2_@PDMS Urinary Catheter

Photothermal characteristics of PDMS, 1T0.1, 1T0.3, 1T0.5, 2H0.1, 2H0.3, and 2H0.5 samples with a 5.08-cm diameter were determined. The photothermal effect was triggered by a near-infrared (NIR) 808-nm laser with different power densities, of 0.5, 1, and 1.5 W/cm^2^ in a TSB solution. The TSB solution was prepared by mixing 6 g of TSB powder in 200 mL of DI water and sterilizing it in the autoclave for 2 h under 1.5 kg/cm^2^ of pressure. Each sample was added to 1.5 mL of the TSB solution in a culture tube and then irradiated for 10 min. The temperature was recorded every 60 min by a thermal imaging camera (TG267, FLIR, Wilsonville, OR, USA), which was connected to a thermocouple cable.

### 4.6. Mechanical Properties of MoS_2_@PDMS Urinary Catheter

Circular-shaped samples of PDMS, 1T0.1, 1T0.3, 1T0.5, 2H0.1, 2H0.3, and 2H0.5 with a diameter of 25 mm were prepared for deformation characterization (shear stress vs. shear strain). A rotational rheometer (MRC302, Anton Paar, Graz, Austria) was equipped with PP25 SN64416 parallel plate geometries. The water bath temperature was 25 °C, and the working temperature was set to 37 °C.

### 4.7. Contact Angle Characterization of MoS_2_@PDMS Urinary Catheter

The water contact angle was used to determine the hydrophobicity of the PDMS, 1T0.1, 1T0.3, 1T0.5, 2H0.1, 2H0.3, and 2H0.5 samples. A sample was placed on a flat surface. A DIGIDROP instrument (GBX Scientific Instrument, Dublin, Ireland) was used to observe the surface tension of the samples. After water was dropped and reached the surface, the contact angle was calculated using WinDrop++ software (GBX Scientific Instrument, Dublin, Ireland, version 4.1).

### 4.8. Bacterial Growth Curve

Twenty microliters of Gram-negative bacteria (*E. coli*) and Gram-positive bacteria (*S. aureus*) were each cultured in 3 mL of a TSB solution for 18 h. Bacterial solutions were placed in an incubator shaker at 170 rpm and 37 °C to acquire an optical density (OD)_600_ of >1.2. Samples were cut in a circular shape with a 1.9-cm diameter, which was put into a bacterial solution with an OD_600_ of 0.2 and then treated with NIR exposure for 10 min. Afterward, the bacterial solution was returned to a shaker incubator (LM-80D, Bioman Scientific, Taipei, Taiwan) at 170 rpm and 37 °C. The OD_600_ value was recorded every 30 min for 4 h to obtain the growth curve characterization. Three replicates (n = 3) were conducted for each antibacterial experiment to ensure reliability and reproducibility of the results. Standard deviation was used to represent the variability in the data as part of the statistical analysis.

### 4.9. Bacterial Colonies in Agar Plates

Agar plates were prepared by mixing LB powder and agar powder in sterilized distilled water and autoclaved for 1.5 h at a pressure of 1.5 kg/cm^2^. The hot agar solution was poured into a Petri dish and then left until it cooled down to form a gel. The PDMS, 1T0.3, and 2H0.3 (5.1-cm diameter) samples were immersed in 1.5 mL of a bacterial solution (*E. coli* and *S. aureus*) with an OD_600_ of 0.2 and then exposed to 1.5 W/cm^2^ of NIR light. The bacterial solution (20 µL) was taken out after irradiation for 0, 5, and 10 min, seeded onto an agar plate, and spread by glass beads for 3 min. The disk was put in an incubator at 37 °C for 24 h. ImageJ software (ImageJ.JS 1.54s) was used to count colonies of bacteria, and the percentage bacterial survival was calculated using Equation (1):

(1) $$Percent\;Bacterial\;Survival\;(\%)=\;\frac{{OD}_{value}(sample)}{{OD}_{value}(control)}\times 100\%$$

### 4.10. Bacterial Morphology Before and After Irradiation

Bacterial morphologies of the PDMS, 1T0.3, and 2H0.3 samples were monitored by examination with SEM before and after NIR light treatment. In brief, 1.9-cm-diameter circular samples were sterilized for 10 min in 70% ethanol. Two milliliters of a bacterial solution with an OD_600_ value of 0.2 was incubated with a sample for 4 h. Samples were divided into two groups: untreated and laser-treated. In the laser-treated group, an NIR laser source was applied to the samples in the bacterial solutions for 5 min after the incubation process. The bacterial solution (1 mL) was transferred to a microcentrifuge for washing twice with DI water, and was then mixed with 4% paraformaldehyde for 30 min. Afterward, it was washed twice with DI water and then mixed with 70% ethanol. Twenty microliters of the bacterial and ethanol solution was spread on silicon glass, then dried for 30 min at 60 °C. Finally, to avoid charging during SEM observation, samples were coated with gold in an ion sputter coater (E-1010, Hitachi, Tokyo, Japan).

### 4.11. Biocompatibility of the PDMS@MoS_2_ Urinary Catheter

To ensure safe interactions between the material and normal cells (Vero cells), an in vitro assay was used to test the biocompatibility of the PDMS, 1T0.3, and 2H0.3 samples. Fresh DMEM supplemented with 10% FBS and 1% antibiotic was prepared as the culture medium. Vero cells (8000 cells) were seeded into six-well plates, cultured with sterilized samples of 15 mm in diameter, and then maintained in an incubator at 37 °C for 24 h under a 5% CO_2_ atmosphere. Subsequently, cells were incubated for 1 h at 37 °C. The growth medium was extracted, followed by two washes of the cells with a 1× PBS solution. Subsequently, 1 mL of 1× PBS was introduced. Vero cell proliferation was observed with fluorescence microscopy. Cells were stained with 1.5 µL of a 1 mM calcein AM solution in the culture medium to detect living cells.

## 5. Conclusions

1T-MoS_2_ NSs were successfully synthesized using a hydrothermal method, and their transformation into semiconductor-phase 2H-MoS_2_ NSs through annealing was confirmed by XRD, XPS, UV-Vis, and Raman spectra. A urinary catheter was developed with varying contents of 1T-MoS_2_ NSs and 2H-MoS_2_ NSs embedded in PDMS. Increasing the MoS_2_ content in PDMS enhanced the photothermal ability and slightly decreased the water contact angle, without altering the mechanical properties. Both 1T-MoS_2_ and 2H-MoS_2_ PDMS urinary catheters effectively achieve bacterial eradication temperatures with 10 min NIR laser irradiation, with higher MoS_2_ concentrations reaching sterilization thresholds more rapidly. For the agar plate test, exposure of 1T0.3 and 2H0.3 to NIR for 10 min provided an excellent antibacterial effect, completely eradicating *E. coli* and killing over 99.9% of *S. aureus*. The hyperthermic effect caused bacterial membrane leakage and even bacterial destruction. The heat generated through photothermal conversion on the metallic and semiconducting phases of the MoS_2_@PDMS urinary catheters shows great promise for the rapid sterilization of bacteria. By integrating phase-controlled 1T/2H MoS_2_ NSs into PDMS, this system advances clinical translation by ensuring durable antibacterial efficacy through bulk embedding and employing a photothermal mechanism to effectively combat Gram-positive and Gram-negative bacteria, while addressing key practical and material requirements for urinary catheter applications.

## Data Availability

The data presented in this study are available upon request from the corresponding authors.

## Supplementary Materials

The following supporting information can be downloaded at: https://www.mdpi.com/article/10.3390/ijms27114806/s1.
