# Supplementary material for: Phase-Dependent MoS2 Nanosheets-Embedded Urinary Catheter for Advanced Photothermal Sterilization
Source: Int J Mol Sci. 2026 May 26;27(11):4806. doi: 10.3390/ijms27114806 (PMC13256306; doi:10.3390/ijms27114806)
Supplement: Supplementary file 1 [file ijms-27-04806-s001.zip › ijms-4262529-supplementary.pdf]

Supporting Information

Phase-Dependent MoS_2 Nanosheets-Embedded Urinary Catheter for Advanced Photothermal Sterilization

Muhammad Saukani [1,†], Chien-Hung Lai [2,3,4,5,†], Dyah Ika Krisnawati [6,7], Hsiu-Yi Chu [8], Andy C. Huang [9,10,*] and Tsung-Rong Kuo [11,12,*]

Department of Mechanical Engineering, Faculty of Engineering, Universitas Islam Kalimantan MAB, Banjarmasin 70124, Kalimantan Selatan, Indonesia; saukani@uniska-bjm.ac.id

[2] Department of Physical Medicine and Rehabilitation, School of Medicine, College of Medicine, Taipei Medical University, Taipei City 110, Taiwan; chlai@h.tmu.edu.tw

[3] Department of Physical Medicine and Rehabilitation, Taipei Medical University Hospital, Taipei City 110, Taiwan

[4] Taipei Neuroscience Institute, Taipei Medical University, Taipei City 110, Taiwan

[5] Graduate Institute of Biomedical Optomechatronics, College of Biomedical Engineering, Taipei Medical University, Taipei City 110, Taiwan

[6] Department of Nursing, Faculty of Nursing and Midwifery, Universitas Nahdlatul Ulama Surabaya, Surabaya 60237, East Java, Indonesia; dyahika@unusa.ac.id

[7] Center for Continuing Care Research (C3R), Universitas Nahdlatul Ulama Surabaya, Surabaya 60237, Indonesia

[8] Graduate Institute of Biomedical Materials and Tissue Engineering, College of Biomedical Engineering, Taipei Medical University, Taipei City 110, Taiwan; d825111001@tmu.edu.tw

[9] Department of Urology, Taipei Medical University Hospital, Taipei City 110, Taiwan

[10] TMU Research Center of Urology and Kidney and Department of Urology, School of Medicine, College of Medicine, Taipei Medical University, Taipei City 110, Taiwan

[11] Graduate Institute of Nanomedicine and Medical Engineering, College of Biomedical Engineering, Taipei Medical University, Taipei City 110, Taiwan

[12] International Ph.D. Program in Biomedical Engineering, College of Biomedical Engineering, Taipei Medical University, Taipei City 110, Taiwan

* Correspondence: 241010@h.tmu.edu.tw (A.C.H.); trkuo@tmu.edu.tw (T.-R.K.)

† These authors contributed equally to this work.

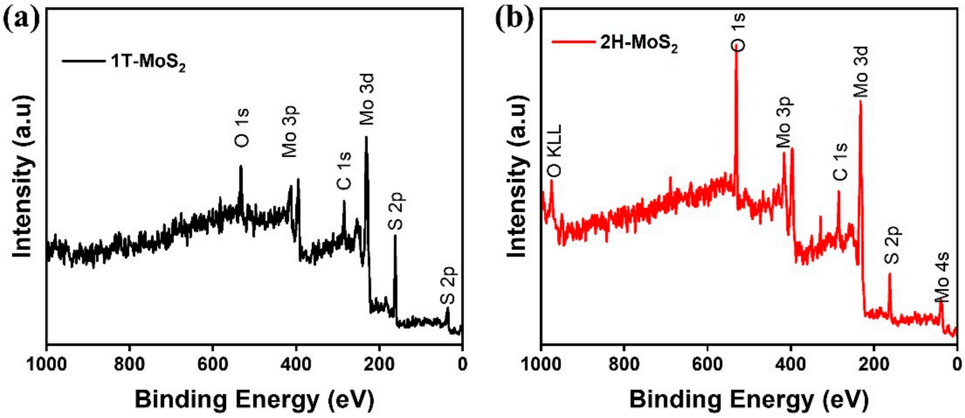

Figure S1. Survey scans of the overall electronic states of (a) 1T-MoS_2 NSs and (b) 2H-MoS NSs.

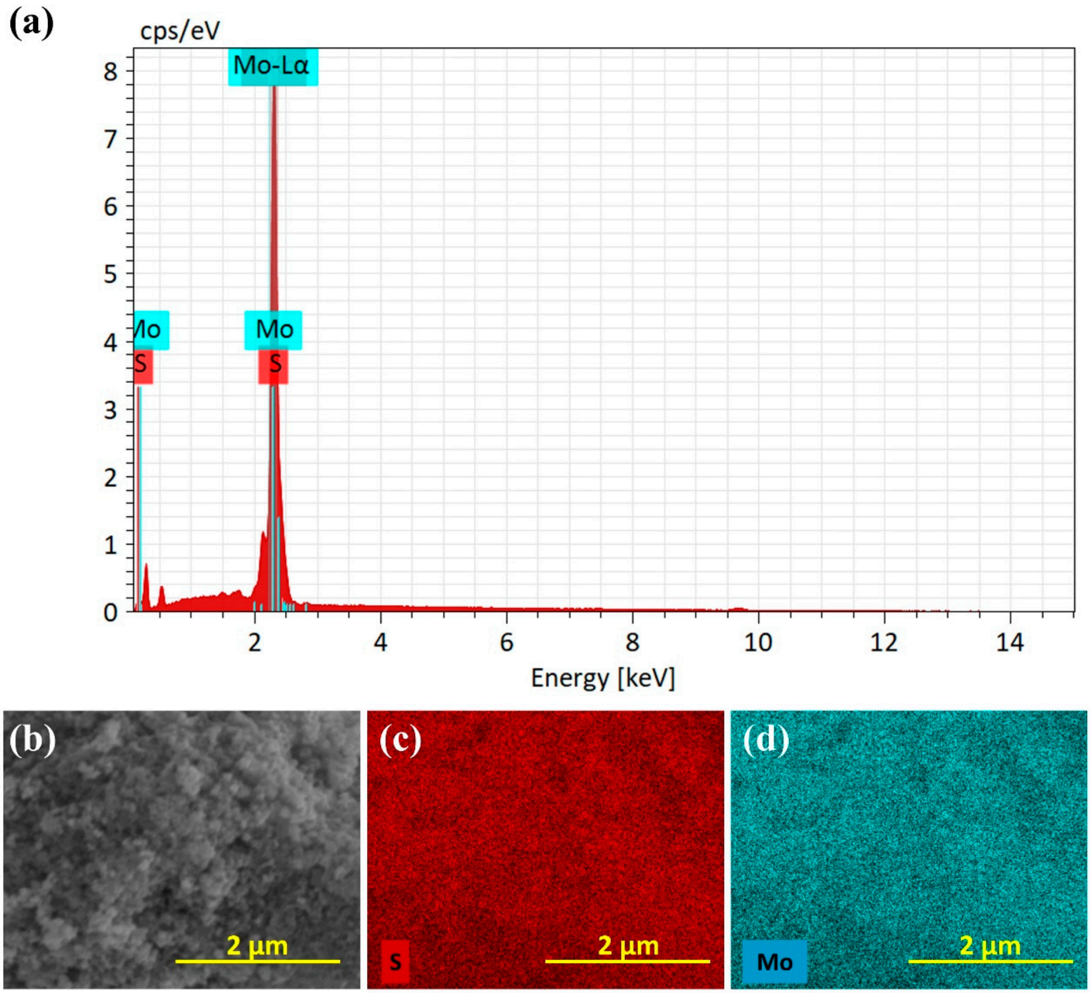

Figure S2. (a) EDX spectrum of 1T-MoS_2 NSs. (b) SEM image of 1T-MoS_2 NSs and its EDX mapping of (c) S and (d) Mo.

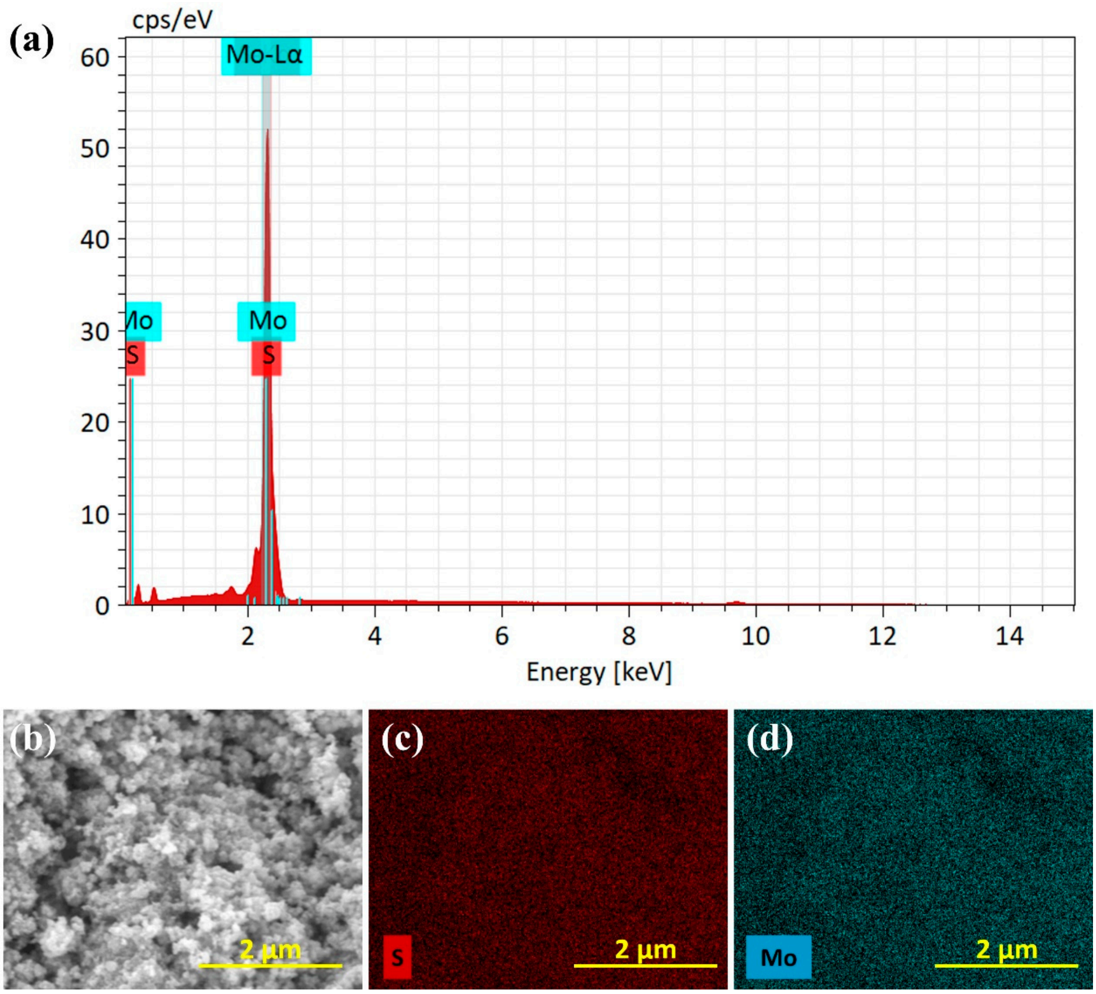

Figure S3. (a) EDX spectrum of 2H-MoS_2 NSs. (b) SEM image of 2H-MoS_2 NSs and its EDX mapping of (c) S and (d) Mo.

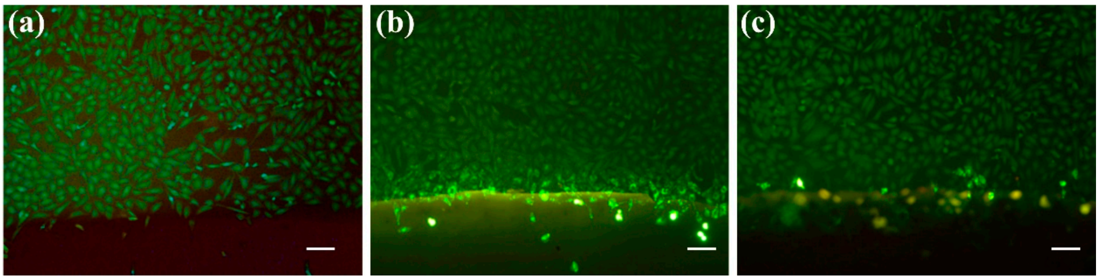

Figure S4. Fluorescence images of Vero cells incubated with (a) PDMS, (b) 1T-MoS_2@PDMS and (c) 2H-MoS_2@PDMS for 24 h. Fluorescence image demonstrated normal cell proliferation after 24 h of culture with PDMS urinary catheter, as indicated by the green-stained Vero cells. Similarly, 1T-MoS_2@PDMS and 2H-MoS_2@PDMS showed comparable cell proliferation after 24 hr. The scale bars are 100 μm.

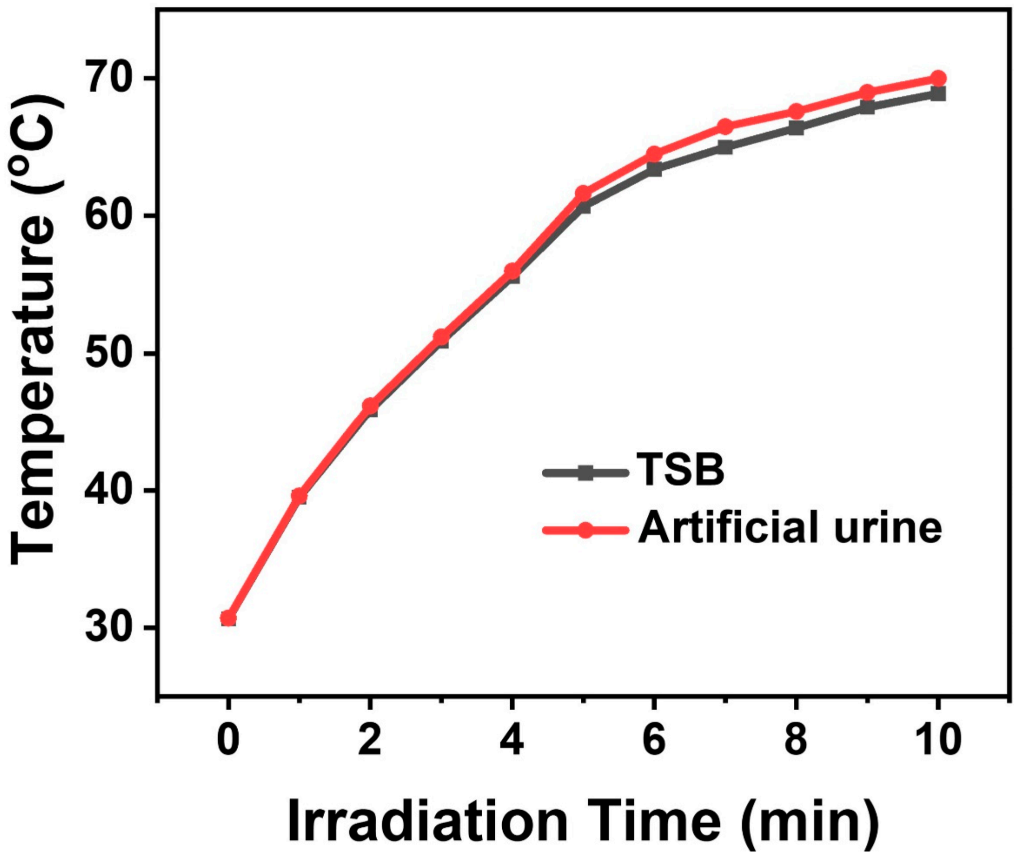

Figure S5. Photothermal performance of the 2H0.5 urinary catheter in artificial urine and the TSB.

Table S1. Elemental composition amounts in 1T-MoS_2 NSs.

Element	Atomic no.	Mass norm. (%)	Atom (%)
Sulfur	16	35.97	62.69
Molybdenum	42	64.03	37.31
Sum		100.00	100.00

Table S2. Elemental composition amounts in 2H-MoS_2 NSs.

Element	Atomic no.	Mass norm. (%)	Atom (%)
Sulfur	16	20.60	61.25
Molybdenum	42	38.99	38.75
Sum		100.00	100.00

Calculation of Photothermal Conversion Efficiency of the MoS_2 NSs-Embedded Urinary Catheters

The photothermal conversion efficiency of the MoS_2 NSs-embedded urinary catheters was evaluated following a previously established approach, with the detailed calculation outlined below:

$$\eta = \frac{hS\,(T_{max} - T_{surr}) - Q_{dis}}{I(1 - 10^{-A_{808}})} \qquad (1)$$

In Equation (1), *h* represents the heat transfer coefficient, *S* denotes the surface area for heat transfer, T_{max} is the equilibrium temperature, and T_{surr} refers to the surrounding environment's temperature. Q_{dis} represents the heat dissipated to the surroundings, *I* is the laser irradiation intensity, and A_{808} is the absorbance of the photothermal material at a wavelength of 808 nm. To determine *hS*, a parameter θ is calculated as follows:

$$\theta = \frac{T - T_{sur}}{T_{max} - T_{surr}} \qquad (2)$$

The time constant of a sample system can be determined using Equation (3).

$$\tau_s = \frac{t}{-\ln(\theta)} \qquad (3)$$

The values of *hS* can be determined using Equation (4).

$$hS = \frac{m_D\, C_D}{\tau_s} \qquad (4)$$

For example, based on above approach, the photothermal conversion efficiency of the 1T01 was calculated. From the experimental design, the T_{surr} was 27.9 °C, the laser intensity is 1.5 W. The absorbance of the 1T01 was 0.2628 at wavelength of 808

nm. From Figure S4a, the T_{max} was 47.8 °C. From Figure S4b, τ_s was calculated to be 300.7 s and then hS was obtained as 13.93 mW/°C. Therefore, the photothermal conversion efficiency of the 1T01 was calculated to be 40.24%. For 1T01, 1T03, 1T05, 2H01, 2H03, and 2H05, photothermal conversion efficiencies were calculated to be 40.24, 45.38, 41.32, 45.35, 43.49, and 42.98, respectively. The related parameters are listed in Table S3.

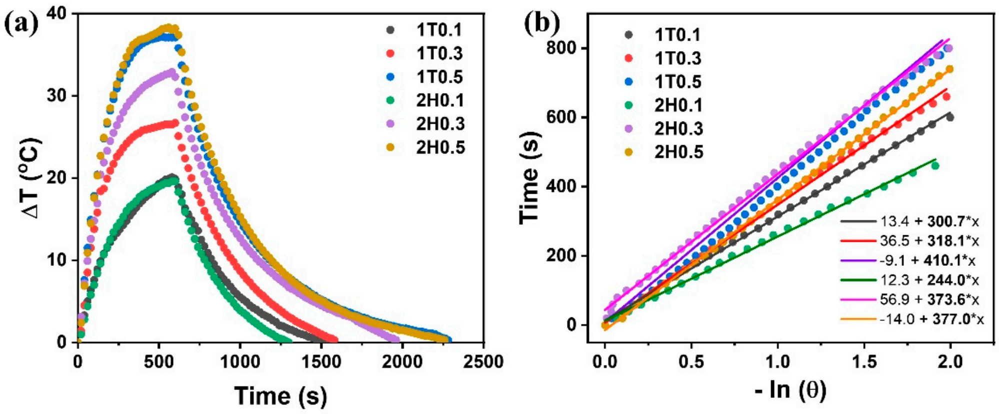

Figure S6. (a) Photothermal curves of 1T01, 1T03, 1T05, 2H01, 2H03, and 2H05 with 808 nm laser irradiation. (b) Linear time data versus -ln (θ) obtained from the cooling period of Figure S5a.

Table S3. Parameters for the calculation of photothermal efficiency.

Sample	τ_s	hS	T_{max}	T_{surr}	ΔT	A_{808}	Efficiency
**1T0.1**	300.7	13.93	47.8	27.9	19.9	0.2628	40.24
**1T0.3**	318.1	13.14	54.9	26.3	28.6	0.3263	45.38
**1T0.5**	410.1	10.19	64.5	25.0	39.5	0.3263	41.32
**2H0.1**	244.0	17.13	48.1	28.9	19.2	0.4491	45.35
**2H0.3**	373.6	11.20	57.8	25.5	32.3	0.3866	43.49
**2H0.5**	377.0	11.08	64.9	24.5	40.4	0.5408	42.98
